# Editorial: Signaling through WD-Repeat Proteins in Plants

**DOI:** 10.3389/fpls.2016.01157

**Published:** 2016-08-03

**Authors:** Marco A. Villanueva, Tania Islas-Flores, Hemayet Ullah

**Affiliations:** ^1^Instituto de Ciencias del Mar y Limnología, Unidad Académica de Sistemas Arrecifales, Universidad Nacional Autónoma de MéxicoPuerto Morelos, Mexico; ^2^Department of Biology, Howard UniversityWashington, DC, USA

**Keywords:** hormonal regulation, heterotrimeric G-protein, plant innate immunity, RACK1, signal transduction, WD-repeat protein, WD40 domain

Plants are sessile organisms that rely on appropriate signal-transduction responses in order to cope with the challenges imposed by their environment, and must be able to recognize potential damage or benefit to respond accordingly. These response mechanisms are mediated by specific sets of signal receptors, effector proteins interacting through scaffolding assemblies, second messengers, and transcription factors, among other components. The specific responses occur through protein–protein interactions conveyed by particular sequence modules such as the WD-repeat (WDR), which has been evolutionarily conserved in many proteins participating in signaling events. This module consists of a sequence that spans a number of conserved amino acids and ends with tryptophan and aspartate (WD) residues (Neer et al., 1994). The WDR proteins comprise a breathtakingly diverse superfamily of regulatory proteins, representing a breadth of biochemical mechanisms and cellular processes (van Nocker and Ludwig, 2003). One particular variant, the WD40 domain, starts with glycine and histidine followed by a 40 amino acid stretch that ends with WD. These domains usually assemble into β-stranded platforms that form a structure called the β-propeller (Wang et al., 2013). Through the interactions on various parts of the platform, signal-transduction molecules are brought in close proximity to relay conformational changes or enzyme-mediated modifications. The topic “*Signaling through WD-repeat proteins in plants*” consists of two comprehensive reviews, a perspective, and two articles of original research on the field that help understand how some of these WDR proteins from plants interact with other molecules in response to particular signals.

The article by Guerriero et al., places in perspective, a potential role of WDR proteins in the biosynthesis and regulation of plant cell wall formation. They propose that, because in plants the formation of lignocellulosic biomass involves extensive synthesis of cell wall polysaccharides, which also requires the assembly of large transmembrane enzyme complexes, intensive vesicle trafficking, interactions with the cytoskeleton, and coordinated gene expression, WDR proteins could participate in these processes. They mention that, by experimental approaches in *Arabidopsis thaliana*, three WDR proteins have been linked to the cell wall, whereas significantly more potential candidates linked to cell wall-related processes in *A. thaliana* have been obtained by *in silico* analysis. They also suggest a relationship based on the frequent observation of WDR and cell wall synthetic gene collinearity. Finally, they discuss the prospects of biotechnological engineering for enhanced biomass production.

The research article by Huang et al., shows that two pathways, HEXOKINASE 1 (HXK1) and Regulator of G-protein Signaling 1 (AtRGS1), mediate D-glucose signaling in *A. thaliana*. They show signaling crosstalk between these pathways, and that the WDR protein AGB1 is the triggering signaling element after G protein-mediated sugar signaling, followed by the downstream action of HXK1 on AtRGS1 signaling. They also show *in planta* interaction between RHIP1and both AtRGS1 and AtHXK1-a requirement for some glucose-regulated gene expression, suggesting a role for RHIP1 in glucose signaling. They conclude that glucose signaling is a complex hierarchical relationship specific to the target gene and sugar phenotype.

Next, the research article by Sabila et al., addresses the mechanism by which specific tyrosine phosphorylation mediates AtRACK1A interactions. By site-directed mutagenesis in combination with the Yeast Split-Ubiquitin Assay and BiFC Analysis, they determined that phosphorylation on tyrosine 248 is a requisite for AtRACK1A homo-dimerization. They also showed that exposing *Arabidopsis* plants to heat stress resulted on such Tyr248 phosphorylation. In addition, yeast cells expressing AtRACK1A WT, but not non-dimerizing AtRACK1A-Y248F grew in the Yeast Split-Ubiquitin Assay when exposed to UV-B light. Their results suggested that AtRACK1A homo-dimerization induces UV-B stress resistance and allows contact with partners interacting on the same surface of AtRACK1A, or recruiting novel partners in newly exposed regions, both unreachable in the monomer.

Finally, two comprehensive reviews cover, in one case, the evolution and role of ternary WD40 repeat-containing protein complexes in plant immunity; and in the other, the various described roles of plant RACK1 in plant signaling. The review article by Miller et al. engages in the review of ternary complexes formed by plant proteins containing WD40 repeats and their role on immunity. They specifically focused on Gβ and TRANSPARENT TESTA GLABRA1 (TTG1) proteins, that are constituents of ternary complexes. These proteins contain seven tandem WD40 repeats and are involved in plant innate immune signaling. Gβ is coupled to type-I membrane receptors while TTG1 to transcription factors whereby functioning at opposite ends of a plant immune signaling pathway. When activated, the heterotrimeric G protein releases the Gβγ dimer to regulate the MAPK cascade by interacting directly with a MAPK protein or by recruiting RACK1 as MAPK scaffold. The authors conclude that Gβ and TTG1 represent the interaction hubs in their specific pathways despite the different evolutionary history of their mechanisms and targets.

Two decades after the first report of the plant homolog of the Receptor for Activated C Kinase 1 (RACK1) in cultured tobacco BY2 cells (Ishida et al., 1993), and as a tribute to this versatile scaffolding WDR protein, Islas-Flores et al. provide an overview of the most recent findings regarding the various interacting partners and roles of this protein in diverse plant processes. The authors discuss its promiscuous properties emphasizing the spatio-temporal regulation of critical signal-transduction events such as hormonal control, stress responses, development, immune defense, protein translation regulation, miRNA production, photosynthesis, and cell wall biogenesis. They relate these processes from the information of at least 138 reported ligands recently identified using the split-ubiquitin based and conventional yeast two-hybrid assays. The authors conclude from the information acquired that, in spite of the high degree of conservation of its structure, the functions of the plant RACK1 homolog appear to be distinct and diverse from those in yeast, mammals, insects, etc. They also conclude that even though there is significant novel information regarding the many functions in which plant RACK1 has been reported to participate, there is still much to learn about the missing ligand partners.

## Author contributions

All authors listed contributed substantially to the work. MV, TI and HU wrote, proofread, and approved the manuscript for publication.

### Conflict of interest statement

The authors declare that the research was conducted in the absence of any commercial or financial relationships that could be construed as a potential conflict of interest.
